# Temporal Learning Among Prefrontal and Striatal Ensembles

**DOI:** 10.1093/texcom/tgaa058

**Published:** 2020-08-29

**Authors:** Eric Emmons, Gabriela Tunes-Chiuffa, Jeeyu Choi, R Austin Bruce, Matthew A Weber, Youngcho Kim, Nandakumar S Narayanan

**Affiliations:** Department of Psychiatry, Yale University, New Haven, CT 06515, USA; Universidade Federal do ABC, Sao Paulo 09210-580, Brazil; School of Dentistry, Pusan National University, Yangsan 50612, Republic of Korea; Department of Neurology, University of Iowa, Iowa City, IA 52242, USA; Department of Neurology, University of Iowa, Iowa City, IA 52242, USA; Department of Neurology, University of Iowa, Iowa City, IA 52242, USA; Department of Neurology, University of Iowa, Iowa City, IA 52242, USA

**Keywords:** corticostriatal, interval timing, learning, prefrontal cortex, striatum

## Abstract

Behavioral flexibility requires the prefrontal cortex and striatum, but it is unclear if these structures play similar or distinct roles in adapting to novel circumstances. Here, we investigate neuronal ensembles in the medial frontal cortex (MFC) and the dorsomedial striatum (DMS) during one form of behavioral flexibility: learning a new temporal interval. We studied corticostriatal neuronal activity as rodents trained to respond after a 12-s fixed interval (FI12) learned to respond at a shorter 3-s fixed interval (FI3). On FI12 trials, we found that a key form of temporal processing—time-related ramping activity—decreased in the MFC but did not change in the DMS as animals learned to respond at a shorter interval. However, while MFC and DMS ramping was stable with successive days of two-interval performance, temporal decoding by DMS ensembles improved on FI3 trials. Finally, when comparing FI12 versus FI3 trials, we found that more DMS neurons than MFC neurons exhibited differential interval-related activity early in two-interval performance. These data suggest that the MFC and DMS play distinct roles during temporal learning and provide insight into corticostriatal circuits.

## Introduction

Behavioral flexibility requires learning to adapt to uncertainty. Two forebrain structures critical for flexibility are the prefrontal cortex and striatum (Fuster 2008; Kehagia et al. 2010). Prefrontal cortical neurons densely innervate the striatum (Gabbott et al. 2005; Wall et al. 2013) and disruptions of either structure profoundly impact the learning of new goals, rules, and strategies (Ragozzino 2007; Hart et al. 2018). Dysfunctional corticostriatal circuits and connectivity are implicated in a range of psychiatric and neurological disorders (Deutch 1993; Shepherd 2013). However, the relative contributions of prefrontal and striatal networks to behavioral flexibility are unclear, and clarifying their respective roles may provide novel approaches to identifying new biomarkers or treatments for human brain diseases.

One task that provides an ideal window into behavioral flexibility is interval timing, which requires participants to estimate an interval of several seconds via a motor response. Across species, interval timing requires the prefrontal cortex and striatum (Matell and Meck 2004; Coull et al. 2011; Merchant and de Lafuente 2014; Mello et al. 2015; Emmons et al. 2016, 2017; Dallérac et al. 2017). Work from our group and others has shown that both prefrontal and striatal neurons encode temporal information via “time-related ramping” activity—or monotonic changes in firing rate over a temporal interval (Donnelly et al. 2015; Narayanan 2016; Bakhurin et al. 2017; Emmons et al. 2017; Kim et al. 2018; Wang et al. 2018). Our past work suggested that ramping activity in neurons of the medial frontal cortex (MFC) and the dorsomedial striatum (DMS) is very similar, with one-third of neurons in each area exhibiting such activity (Emmons et al. 2017). We have also found that MFC inactivation attenuates DMS ramping (Emmons et al. 2017, 2019) and that corticostriatal stimulation is sufficient to recover decreases in DMS ramping caused by MFC inactivation (Emmons et al. 2019). These data suggest that DMS ramping is closely linked to MFC ramping and suggest the hypothesis that MFC and DMS ensembles respond similarly as animals learn new temporal intervals. By contrast, recordings from primate lateral prefrontal cortex and caudate indicate that striatal ensembles encode stimulus-response associations earlier than prefrontal ensembles, leading to the hypothesis that prefrontal and striatal ensembles play distinct roles during learning (Pasupathy and Miller 2005; Histed et al. 2009; Antzoulatos and Miller 2011).

We tested if MFC and DMS ensembles played similar or distinct roles during temporal learning. We recorded MFC and DMS activity in rodents as they learned to respond to a new temporal interval. Animals trained in the temporal context of one 12-s interval learned a new temporal context where two intervals—either 12 s or 3 s—could be presented. We report three main results. First, for 12-s intervals, MFC ramping decreased in the context of two-interval sessions compared to one-interval sessions, whereas DMS ramping did not change. Second, while MFC and DMS ramping was stable over subsequent days of two-interval performance, DMS ensembles improved temporal decoding for 3-s intervals. Finally, comparing activity on 12-s versus 3-s intervals revealed that DMS neurons were more likely to have different activity patterns compared to MFC neurons, particularly on the first day of two-interval performance. These data suggest that the MFC and DMS play distinct roles during temporal learning.

## Materials and Methods

### Rodents

**Figure 1:**
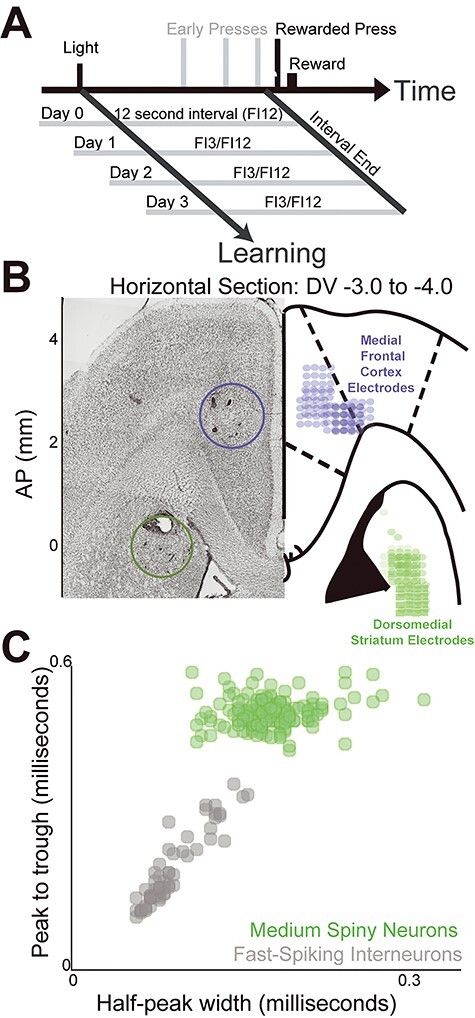
Fixed-interval timing tasks and recording locations. (*A*) On Day 0, rodents performed fixed-interval timing tasks in which a reward was given for the first lever press after a 12-s interval (FI12). Interval start was cued by a house light, motivation was a liquid reward, and presses before interval end were not reinforced. On Day 1, there was a new temporal context with the addition of a second, shorter 3-s interval (FI3) cued by a distinct light. FI3 trials were randomly intermixed with FI12 trials. Recordings were performed for 3 days of two-interval performance (Days 1–3). (*B*) Animals were implanted with multielectrode recording arrays targeting the MFC and DMS; horizontal sections with approximate AP coordinates shown. (*C*) MSNs within the DMS were identified based on waveform shape.

All procedures were approved by the University of Iowa IACUC, and all methods were performed in accordance with the relevant guidelines and regulations (protocol #7072039). Seven male Long-Evans rats were trained on the 12-s fixed-interval timing task (FI12) (Emmons et al. 2016, 2017). In this task, rats press the lever in anticipation of the end of the interval. We have used fixed-interval timing to study corticostriatal circuits in detail in rodents (Narayanan et al. 2012; Parker et al. 2014; Parker, Ruggiero, et al. 2015; Emmons et al. 2016, 2017; Kim et al. 2017; Parker et al. 2017; Emmons et al. 2019, 2019) and in humans (Parker, Chen, et al. 2015; Kelley et al. 2018). Rats were motivated by water-restriction maintained at 85–90% of their free access weight. Food was freely available. In brief, the rats were autoshaped to press a lever for water reward using a fixed-ratio task before being trained on the 12-s fixed-interval timing task (FI12). Trials began with the presentation of a house light, and the first response made after 12 s resulted in the delivery of a water reward, a concurrent click, and termination of the house light (Fig. 1*A*). Responses made before the interval ended were not reinforced. Trials were separated by a randomly chosen 6-, 8-, 10-, or 12-s intertrial interval. After animals behaved consistently, the MFC and DMS were each implanted with recording electrodes (Fig. 1*B*; see below). Animals were then acclimatized to the recording procedures and recordings were made during behavior in the one-interval task (FI12 trials only; Day 0). The following day, an additional 3-s interval (FI3) was added to the task and cued by a light distinct from the one used to indicate FI12. In general, sessions were 60 min. Critically, task stimuli on FI12 trials were identical between one-interval and two-interval sessions. FI12 and FI3 trials were randomly intermixed. Behavior and simultaneous neuronal activity in the MFC and DMS were recorded over the following 3 days of two-interval performance (FI12/FI3 trials on Day 1, Day 2, and Day 3). Data from subsequent two-interval recording sessions in 5/7 rodents were included in prior manuscripts (Emmons et al. 2016, 2017).

#### Surgical and Histological Procedures

Animals were anesthetized using ketamine (100 mg/kg IP) and xylazine (10 mg/kg IP), and a surgical level of anesthesia was maintained using ketamine supplements (10 mg/kg IP). Craniotomies were drilled above the left MFC and left DMS and 4 holes were drilled for skull screws, which were connected to electrode recording arrays via a separate ground wire. Microelectrode arrays were composed of 4 × 4 50-μm stainless steel wires (250 μm between wires and rows). These arrays were positioned in the MFC (coordinates from bregma: AP +3.2, ML ±1.2, DV -3.6 @ 12° in the anterior plane) and the DMS (coordinates from bregma: AP +0.0, ML ±4.2, DV -3.6 @ 12° in the posterior or lateral plane) while recording neuronal activity to verify that implantation was in correct brain area. The craniotomy was sealed with cyanoacrylate (“SloZap,” Pacer Technologies, Rancho Cucamonga, CA), and the reaction was accelerated by “ZipKicker” (Pacer Technologies) and methyl methacrylate (AM Systems, Port Angeles, WA). Rats recovered for 1 week before being acclimatized to behavioral and recording procedures.

Following these experiments, the rats were anesthetized and sacrificed by injection with 100 mg/kg sodium pentobarbital and transcardially perfused with 4% formalin. Brains were postfixed in a solution of 4% formalin and 20% sucrose before being horizontally sectioned on a freezing microtome. Brain slices were mounted on Superfrost Plus microscope slides and stained for cell bodies using either DAPI or Cresyl violet. Histological reconstruction was completed using postmortem analysis of electrode placement by slide-scanning fluorescent microscopy (Olympus, Center Valley, PA).

#### Neurophysiological Recordings and Neuronal Analyses

Neuronal ensemble recordings were made using a multielectrode recording system (Plexon, Dallas, TX). In each animal, one electrode without single units was reserved for local referencing, yielding 15 electrodes per animal. Offline Sorter (Plexon) was used to analyze the signals after the experiments and to remove artifacts. Spike activity was analyzed for all cells that fired at rates above 0.1 Hz. Principal component analysis (PCA) and waveform shape were used for spike sorting. Single units were defined as those 1) having a consistent waveform shape, 2) being a separable cluster in PCA space, and 3) having a consistent refractory period of at least 2 ms in interspike interval histograms. As in our past work, putative cortical interneurons were excluded at initial spike-sorting because they were difficult to definitively identify (Narayanan and Laubach 2009; Emmons et al. 2017). Putative DMS medium spiny neurons (MSNs) were further separated from striatal interneurons based on waveform peak-to-trough ratio and the half-peak width (Fig. 1*C*; Berke, 2011). All neuronal analyses focused on putative pyramidal neurons and MSNs. Counts of recorded neurons are in Table 1, note that the same electrodes are recorded from across days.

**Table 1:** Neurons recorded in each animal

		Number of neurons		
	Animal	MFC	STR	Totals
Day 0	1	7	9	
	2	7	9	
	3	15	14	
	4	11	8	
	5	3	4	
	6	6	8	
	7	10	15	
Day 1	1	5	5	
	2	7	9	
	3	8	9	
	4	5	2	
	5	3	5	
	6	6	8	
	7	13	20	
Day 2	1	7	4	
	2	6	7	
	3	7	8	
	4	6	3	
	5	4	3	
	6	6	8	
	7	11	21	
Day 3	1	10	6	
	2	6	11	
	3	11	13	
	4	9	4	
	5	4	8	
	6	10	11	
	7	14	27	
Two-interval total		158	192	350
All sessions total		217	259	476

#### Statistics

All data and statistical approaches were reviewed by the Biostatistics, Epidemiology, and Research Design (BERD) Core at the Institute for Clinical and Translational Sciences at the University of Iowa. We used generalized linear-mixed effects models (GLMM; *fitglme.m* in MATLAB, Natick, MA) where the outcome variable was response time. For FI12 trials, the predictor variable was temporal context (i.e., one-interval sessions on Day 0 when FI12 trials were presented alone, or two-interval sessions on Days 1–3 when FI3 trials were presented along with FI12 trials). For two-interval performance, response time on FI12 or FI3 trials was the outcome variable, and Day (Days 1–3) was the predictor variable. Animal-specific variance was included as a random effect; this explicitly accounts for variability between individual animals. For two-interval trials, single-trial analyses were used to find start times and coefficients of variation for FI3 and FI12 trials (Church et al. 1994). Finally, because we cannot definitively track neurons between days, all statistical analyses assumed that each population of neurons was statistically independent.

Analyses of neuronal activity and basic firing properties were carried out using NeuroExplorer (Nex Technologies, Littleton, MA) and custom routines for MATLAB, as described in detail previously (Parker et al. 2014; Emmons et al. 2017, 2019). Peri-event rasters and peri-event time histograms (PETHs) were constructed around houselight and lever press. As in our past work, neuronal ensemble modulations were quantified via PCA, a data-driven set of orthogonal basis vectors that captures patterns of activity in multivariate neuronal ensembles (Chapin and Nicolelis 1999; Narayanan and Laubach 2009). PCA was calculated from average PETHs computed from kernel-density estimates (*ksdensity.m*; FI3: bandwidth 0.2; FI12: bandwidth 0.5) and normalized by Z-score. Notably, single neurons can either ramp up or down, and our past work has not identified reliable differences between these populations (Parker et al. 2014; Parker, Ruggiero, et al. 2015; Narayanan 2016; Emmons et al. 2017; Kim et al. 2017). Consequently, we used absolute values of PC1 scores (indicated by |score|) to compare ramping strength across areas and days as in our past work (Parker et al. 2014, 2017; Emmons et al. 2017; Kim et al. 2017). For time-related ramping, we used GLMMs where PC1 |score| was used as an outcome variable; predictor variables were brain area (MFC vs. DMS) and either temporal context (FI12 on Day 0 vs. FI12/3 sessions on Days 1–3) or day of two-interval performance (Days 1–3). Animal-specific variance was included as a random effect; as with behavioral data, this explicitly accounts for variability between individual rodents. Post hoc testing was performed via estimated marginal means (*emmeans* in R) with Tukey’s method for family-wise comparisons after linear-mixed effects modeling (*lmer* in R).

We also used GLMMs to analyze neuronal modulations. We calculated trial-by-trial GLMMs for all trials and neurons where the outcome was firing rate binned at 0.1 s, the predictors were area (MFC or DMS) or Day 0 or Day 1, and random effects were responses and neurons. Our past work has demonstrated that GLMMs account for the contribution of lever-press response-related activity to time-related ramping; this analysis appropriately considers variance across neurons and animals (Narayanan and Laubach 2009; Emmons et al. 2017, 2019). To examine interval-related modulation for all trials and neurons, we used an equation where the outcome was firing rate, the predictors were interval (FI3 or FI12), area, or day, and random effects were lever-press responses and neurons. Poisson distributions were used for all firing-rate models.

We used a naïve Bayesian classifier to examine neuronal ensemble decoding, as we have in our past work (Emmons et al. 2017; Kim et al. 2017). We calculated kernel density estimates (bandwidth: 1.2) of trial-by-trial firing rates from MFC and DMS neurons. To prevent edge effects that might bias classifier performance, we included data from 6 s prior to trial start and 6 s after interval end. We used leave-one-out cross-validation to predict objective time from firing rate within a trial. We evaluated classifier performance by computing the R^2^ of objective time and predicted time only for bins during the interval. With perfect classification, the R^2^ would approach 1. Classifier performance was compared to ensembles with time-shuffled firing rates. For each area and interval, the performance was quantified via GLMMs of R^2^ versus each day.

## Results

**Figure 2:**
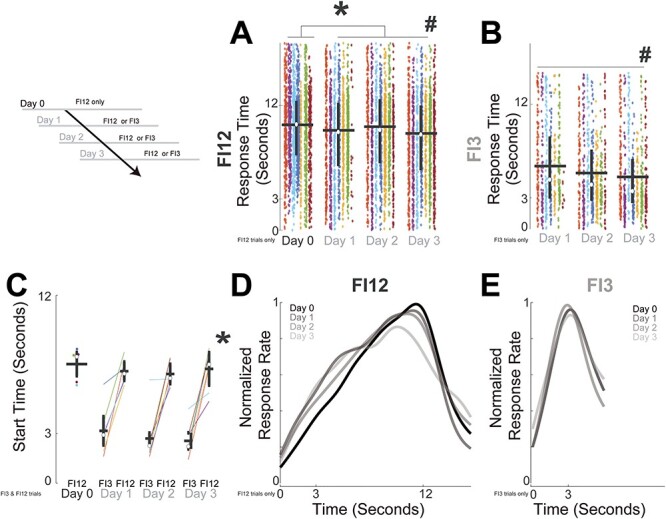
Response times reflect temporal context. Rats trained to respond at one 12-s interval on Day 0 were then trained on Days 1–3 in a new temporal context with two intervals: 12 s (FI12) and 3 s (FI3). (*A*) Plot of all responses from all animals for FI12 trials only on Day 0 and Days 1–3; each animal is plotted in a different color. * indicates a main effect of context (one vs. two intervals, or Day 0 vs. Days 1–3), # indicates a main effect of day (over Days 1–3) via GLMMs. White dots denote the median, horizontal lines denote the mean and vertical lines span the interquartile range. (*B*) Plot of all responses from all animals for FI3 trials only on Days 1–3, # indicates a main effect of day via GLMMs. (*C*) Plot of start times calculated from single-trial analysis for each animal on Days 0–3 for FI3 and FI12 trials; * indicates a main effect of interval via GLMMs. Normalized time-response histograms for (*D*) FI12 trials and for (*E*) FI3 trials from Day 0 (black) and Days 0–3 (shades of gray). Kernel-density histograms for plotting only calculated at 0.1 s bins with a bandwidth of 1 then averaged and normalized across 7 animals. Data from 4799 FI12 responses and 1744 FI3 responses in 7 animals.

We studied behavioral flexibility in the MFC and DMS by introducing a new 3-s fixed interval (FI3) to rats after they had been trained on a task with 12-s fixed intervals (FI12; Fig. 1*A*). On Day 0, animals were presented only with one FI12 interval, but on Days 1, 2, and 3, animals were presented with a new temporal context in which either FI12 or FI3 intervals could be presented. For FI12 trials, response times were significantly longer on Day 0 with only one interval (FI12; 10.14 ± 0.09 s; mean ± SEM) compared to Days 1–3 when the temporal context included two intervals (FI12 or FI3 could be presented, FI12 response times: Day 1: 9.61 ± 0.12 s, Day 2: 9.96 ± 0.13 s, Day 3: 9.31 ± 0.12 s; main effect of context on response time; one-way ANOVA of GLMM: *F*_(7022)_ = 18.3, *P* = 0.00002; Fig. 2*A*). FI12 response times grew shorter over subsequent days of two-interval performance (main effect of Days 1–3 on FI12 response time: one-way ANOVA of GLMM: *F*_(4796)_ = 7.0, *P* = 0.0009; FI12 trials only; Fig. 2*A*). On these days, we also found that FI3 response times shortened over subsequent days of two-interval performance (Day 1: 6.05 ± 0.18 s, Day 2: 5.38 ± 0.15 s, Day 3: 5.01 ± 0.14 s; main effect of Days 1–3 on FI3 response time; one-way ANOVA of GLMMs: *F*_(1741)_ = 8.4, *P* = 0.0002; FI3 trials only; Fig. 2*B*). On the very first day of two-interval performance (Day 1), there was a main effect of FI12 versus FI3 on response time (two-way ANOVA of GLMM: *F*_(2072)_ = 78.8, *P* = 2e^−18^) but no reliable effect of trial order within-session on response time (two-way ANOVA of GLMM: *F*_(2072)_ = 0.9, *P* = 0.34) and no reliable higher interactions (*F*_(2072)_ = 1.4, *P* = 0.23). This analysis suggested that on Day 1, response times did not consistently change over the session for either the FI3 or FI12 trial type.

We analyzed fixed-interval behavior using single-trial analysis, which was developed for peak-interval timing but can be useful to analyze start times during fixed-interval tasks (Church et al. 1994; Emmons et al. 2019). On Day 1, single-trial analysis revealed that animals had earlier start times on FI3 trials compared to FI12 trials (two-way ANOVA of GLMMs of interval and day on start times; main effect of interval: *F*_(43)_ = 10.4, *P* = 0.002; Fig. 2*C*; note that one animal on Day 1 did not have enough FI3 trials for analysis). There was no effect of day or interaction. One indication that timing processes are scalar is that the coefficient of variation (CV—the ratio of the standard deviation of temporal estimates to the mean) is relatively constant at different intervals (Gibbon et al. 1984; Rakitin et al. 1998). Accordingly, we found that during fixed-interval performance, start-time CVs were similar on FI3 and FI12 trials across Days 0–3 (two-way ANOVA of GLMMs of interval and day on CV: main effect of interval: *F*_(43)_ = 0.2, *P* = 0.62; Bayes Factor = 5.9; there were no higher effects of day or interaction). These data suggest that start times during fixed-interval timing could exhibit scalar properties (Gibbon et al. 1984) and demonstrate that during FI12 and FI3 trials animals were guiding their responses in time (Fig. 2*D* and *E*).

**Figure 3:**
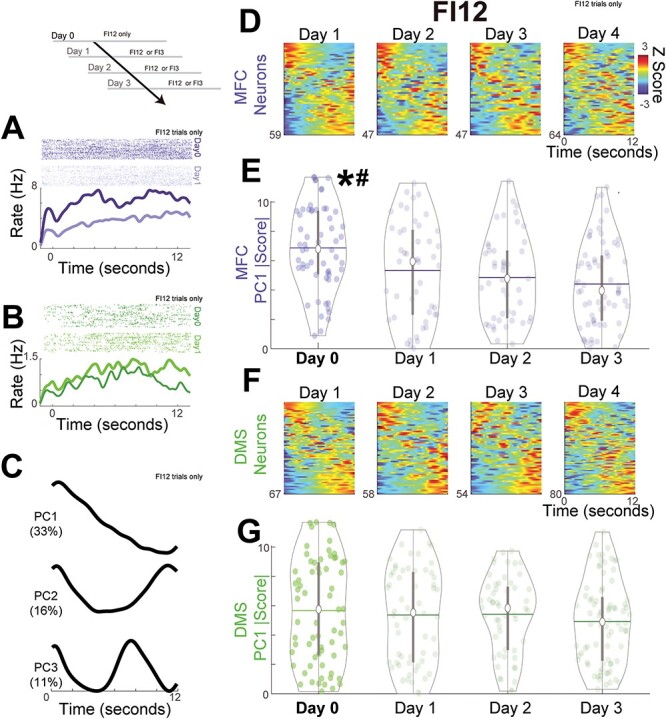
MFC ramping reflects temporal context, but DMS ramping does not. Peri-event rasters for single neurons in the (*A*) MFC (blue) and (*B*) DMS (green). Top panels: each row represents a trial; each tick is an action potential; darker colors represent Day 0 (FI12) and lighter colors represent Day 1 (FI12/FI3); all data are from FI12 trials only. (*C*) PCA revealed 3 main components; percentage of variance is indicated in parentheses. These principal components were calculated from all recordings (MFC and DMS ensembles on Days 0–3). (*D*) PETHs for FI12 trials from MFC sorted by PC1; each row is Z-scored firing rate from each neuron over the 12-s interval. (*E*) PC1 |scores| for MFC for Day 0 (dark blue) and Days 1–3 (light blue). Each circle represents the PC1 |score| from a single neuron; white dots denote the median, horizontal lines denote the mean, and thicker vertical lines span the interquartile range. * indicates an interaction via a two-way ANOVA of GLMMs of PC1 |score| on context (Day 0 vs. Days 1–3) and area (MFC vs. DMS); # indicates *P* < 0.05 of Day 0 vs. Days 1–3 via post hoc testing for MFC. (*F*) PETHs and (*G*) PC1 |scores| from the DMS for Day 0 (dark green) and Days 1–3 (light green). Data from 476 neurons in the MFC and DMS in 7 animals on FI12 trials only.

We recorded neuronal ensembles simultaneously in the MFC and DMS as animals trained on a single interval learned to respond at two intervals (Table 1). As in our past work, we found that neurons in both brain regions exhibited time-dependent ramping, that is, monotonic increases or decreases in firing rate across the interval (Emmons et al., 2017; Fig. 3*A* and *B*). We turned to PCA to compare time-dependent ramping activity as animals learned to perform two-interval tasks (Chapin and Nicolelis 1999; Narayanan and Laubach 2009; Emmons et al. 2017). Consistent with our prior work, we found that principal component 1 (PC1) exhibited time-related ramping (Fig. 3*C*; Emmons et al., 2017; Kim et al., 2017; Parker et al., 2015b, Parker et al. 2014; Zhang et al., 2019). Note that this past work has found no reliable differences between neurons that ramp up or down; hence, we focused on the absolute value of PC1 |score| as a measure of ramping strength (Parker et al. 2014; Narayanan 2016; Emmons et al. 2017).

We compared PC1 |scores| across all areas and days (Days 0–3) via GLMMs on FI12 trials only. Critically for our hypothesis, we found a main effect of temporal context for PC1 |scores| on FI12 trials (one-interval vs. two-interval sessions, or Day 0 vs. Days 1–3; two-way ANOVA of GLMMs: *F*_(472)_ = 12.2, *P* = 0.0005; FI12 trials only) and area (MFC vs. DMS; *F*_(472)_ = 4.5, *P* = 0.03), as well as an interaction between context and area (*F*_(472)_ = 6.5, *P* = 0.01; Fig. 3*D* and *E*). Note that this analysis explicitly included animals as a random effect, accounting for variance contributed by each animal. Post hoc testing revealed that MFC PC1 |scores| were stronger for one-interval compared to two-interval sessions for FI12 trials (*P* = 0.001 Tukey’s post hoc; Fig. 3*E*). However, DMS PC1 |scores| were similar in all sessions (*P* = 0.70 Tukey’s post hoc; Fig. 3*F*–*G*). Critically, there was a main effect of context on PC1 even when PCA was calculated on matched responses (one-interval vs. two-interval sessions—two-way ANOVA of GLMMs only on trials with responses between 11–13 s: *F*_(472)_ = 11.4, *P* = 0.0008). These data imply that differences in PC1 between one-interval and two-interval sessions were not a function of differences in response rate.

Interestingly, for one-interval sessions on Day 0, PC1 |scores| were not different between MFC and DMS (*P* = 0.11 Tukey’s post hoc). This observation was held for trial-by-trial analysis of firing rate on FI12 trials, which revealed a highly significant interaction between time-related ramping, temporal context, and brain area (three-way ANOVA of GLMMs; *F*_(4 045 080)_ = 226.8, *P* = 3e-51; Table 2). Of note, these GLMMs accounted for a variance from each animal and linked neuronal activity to behavior on a trial-by-trial level (Emmons et al. 2017, 2019). Taken together, our data demonstrate that time-related ramping in the MFC but not the DMS decreased in two-interval sessions compared to one-interval sessions. Our results suggest that MFC but not DMS ensembles are sensitive to the temporal context (Jazayeri and Shadlen 2010; Antzoulatos and Miller 2011; Shi et al. 2013).

**Table 2:** Firing rate on FI12 trials in different contexts: one-interval versus two-interval sessions

Predictor	*F*	*P*	
Times	109.3	**1.4E-25**	
Area	6.4	**0.01**	
Learning	2.9	0.09	
Times : area	685.9	**3.5E-151**	
Times : context	135.9	**2.10E-31**	
Area : context	4.0	0.04	
Times : area : context	226.8	**3.0E-51**	

Note: Significant effects are shown in bold. Model: FiringRate~Times × Area × Context+(1|Response)+(1|Neurons). Obs 4 045 080 Model R^2^ 0.18.

**Figure 4:**
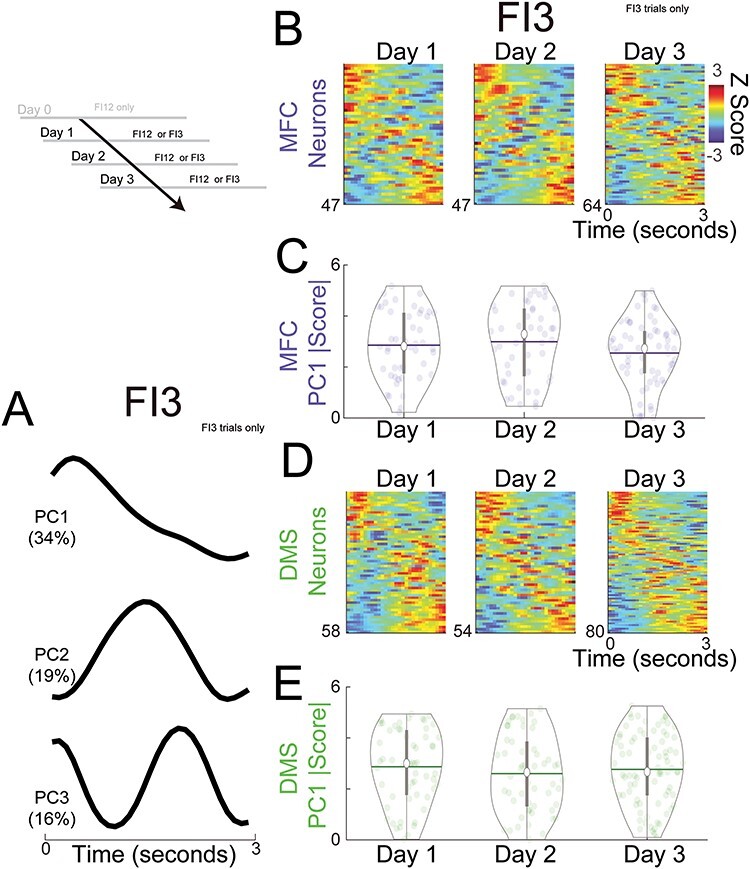
MFC and DMS ramping is stable across Days 1–3 of two-interval performance. (*A*) Principal components for FI3 trials; percentage of variance is indicated in parentheses. These principal components were calculated from all recordings (MFC and DMS ensembles on Days 1–3). (*B*) PETHs for FI3 trials from MFC sorted by PC1. (*C*) PC1 |scores| for MFC on Days 1–3 (light blue). (*D*) PETHs and (*E*) PC1 |scores| from the DMS on Days 1–3 (light green). Data from 350 neurons in the MFC and DMS in 7 animals on FI3 trials only.

Next, we compared PC1 |scores| on subsequent days of two-interval performance for FI12 trials. For FI12 trials, PC1 |scores| did not reliably change across Days 1–3 for either MFC or DMS (Fig. 3*D*–*G*; two-way ANOVA of GLMMs; day: *F*_(413)_ = 0.0, *P* = 0.94; area: *F*_(413)_ = 1.0, *P* = 0.31; interaction: *F*_(413)_ = 0.2, *P* = 0.64). Furthermore, we examined PC1 |scores| on FI3 trials. As in our prior work, PCA revealed highly similar components for FI3 relative to FI12 trials, and PC1 |scores| on FI3 trials also identified time-related ramping (compare Fig. 4*A* to Fig. 3*A*; see Table 3 for percentage of ramping neurons across days; Emmons et al. 2017). For FI3 trials, there were no significant changes in PC1 across Days 1–3 for either area (two-way ANOVA of GLMMs: *F*_(346)_ = 0.4, *P* = 0.52; area: *F*_(346)_ = 1.1, *P* = 0.30; interaction: *F*_(346)_ = 0.6, *P* = 0.45; FI3 trials only; Fig. 4*B*–*E*). Unlike our results for temporal context, these data demonstrate PC1 was remarkably stable across successive days of two-interval performance.

**Table 3:** Percentages of ramping neurons for each interval and day

	Day 0 (%)	Day 1 (%)	Day 2 (%)	Day 3 (%)
MFC—FI3		23	28	17
DMS—FI3		31	20	29
MFC—FI12	59	43	30	31
DMS—FI12	48	55	50	40
MFC—Both		15	15	11
DMS—Both		19	11	16

**Figure 5:**
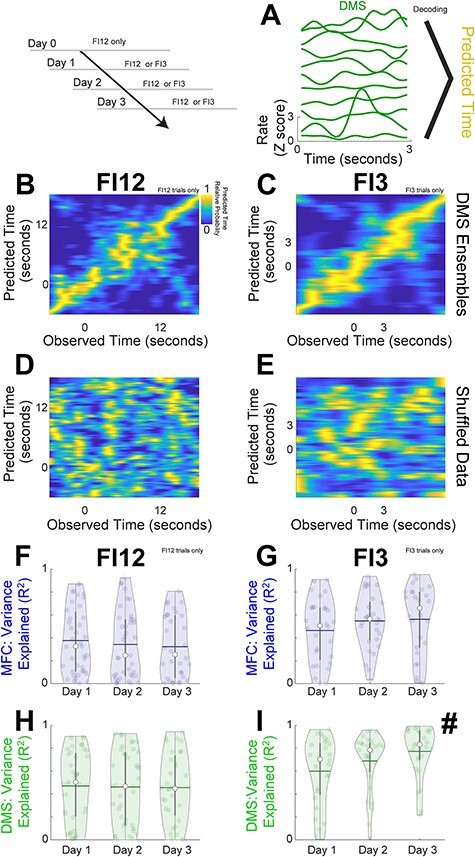
DMS improves temporal decoding with subsequent two-interval performance. (*A*) We trained decoders (naïve Bayesian classifiers) to predict time from firing rate on a trial-by-trial basis. (*B*) Decoder performance for DMS ensembles for FI12 trials and (*C*) for FI3 trials. Predicted time is on the *y*-axis and observed time is on the *x*-axis with the relative probability represented in color and yellow representing the highest relative probability. Decoded performance for the same DMS ensembles with time-shuffled data for (*D*) FI12 trials and (*E*) FI3 trials. We measured decoder performance by calculating the variance explained (R^2^) of predicted versus observed time. R^2^ values plotted for Days 1–3 for MFC on (*F*) FI12 trials and for (*G*) FI3 trials, and for the DMS for (*H*) FI12 trials and for (*I*) FI3 trials. For FI3 trials, R^2^ of DMS ensembles increased with subsequent days of two-interval performance. Each circle represents a single decoded trial for each ensemble; white dots denote the median, horizontal lines denote the mean, and vertical lines span the interquartile range. # indicates a main effect of day in GLMMs. Data from the same 350 MFC and DMS neurons across Days 1–3 in 7 animals as in Fig. 4.

We turned to decoding analyses based on machine learning to capture more complex features of MFC and DMS ensembles beyond time-related ramping (Gouvea et al. 2015; Paton and Buonomano 2018). Specifically, we constructed neuron-dimensional arrays of smoothed trial-by-trial firing rates over the interval binned at 0.1 s (Fig. 5*A*). As in our past work, we decoded time in the interval from ensemble firing rates using naïve Bayesian classifiers. Classifier performance was assessed by computing the variance explained (R^2^) of predicted versus observed time (Emmons et al. 2017; Kim et al. 2017). For all sessions, R^2^ was much less for time-shuffled ensembles—that is, ensembles constructed from neuronal activity shuffled in time (Fig. 5*B*–*I*; comparing shuffled vs. nonshuffled signrank R^2^: *P* = 4 × 10^−47^; Cohen’s d = 1.37). We found that temporal decoding had a main effect of day only for DMS ensembles on FI3 trials (Fig. 5*F*–*I*; one-way ANOVA of GLMMs of day on R^2^: FI3 trials only; *F*_(81)_ = 6.1, *P* = 0.02). These results suggest temporal decoding improved only for DMS ensembles on FI3 trials and are consistent with prior studies that show striatal decoding can be more reliable than cortical decoding (Bakhurin et al. 2017).

**Figure 6:**
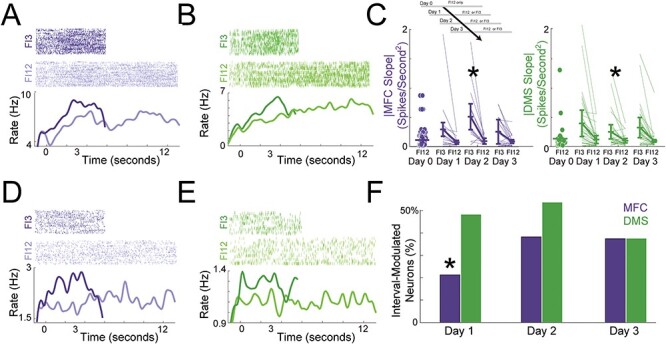
MFC and DMS activity is distinct on FI3 versus FI12 trials. (*A*) An exemplar ramping neuron from the MFC and (*B*) from the DMS; the slope of firing rate versus time was steeper on FI3 versus FI12 trials. (*C*) |slopes| for MFC (blue) and DMS (green) neurons on Day 0, Day 1, Day 2, and Day 3 for FI3 and FI12 trials; for both MFC and DMS, observed |slopes| were higher on FI3 trials; horizontal lines are means and vertical lines are interquartile ranges. * indicates main effect of interval via two-way ANOVA (day and interval) of GLMMs. (*D*) An exemplar neuron from the MFC and (*E*) from the DMS that fired differentially on FI3 versus FI12 trials. (*F*) The number of neurons with a main effect of firing rate versus interval for the MFC and DMS; * indicates *P* < 0.05 via a Chi-squared test. Data from the same 350 MFC and DMS neurons across Days 1–3 in 7 animals as in Fig. 4 on FI12 and FI3 trials.

We directly compared neuronal activity in the MFC and DMS on FI3 and FI12 trials. First, ramping neurons can have distinct slopes of firing rate versus time on FI3 and FI12 trials (Fig. 6*A*–*C*; Table 4). We ran GLMMs where firing-rate slope versus time was the outcome variable, interval and day were predictor variables, and neuron-specific variance was a random effect. As with PC1, we were interested in the slope magnitude rather than sign, so we focused on absolute value (|slope|). Consistent with past work by our group and others, ramping neuron |slopes| were consistently steeper on FI3 than on FI12 trials for both the MFC (Fig. 6*C*; two-way ANOVA of GLMMs of interval and day on MFC |slope|; main effect of interval: *F*_(139)_ = 4.5, *P* = 0.04) and for the DMS (Fig. 6*C*; main effect of interval: *F*_(210)_ = 6.9, *P* = 0.01; Xu et al. 2014; Mello et al. 2015; Emmons et al. 2017; Wang et al. 2018). There was no effect of day or higher interactions for either MFC or DMS. Taken together, these data are consistent with drift-diffusion models of interval timing, suggesting that drift rates increase with shorter intervals (Simen et al. 2011).

**Table 4:** Firing rate on FI3 versus FI12 trials in two-interval sessions

Predictor	*F*	*P*
Area	0.5	0.49
Day	0.6	0.45
Interval	3.9	**0.05**
Area : day	0.8	0.37
Area : interval	0.3	0.57
Day : interval	13.2	**0.0003**
Area : day : interval	6.4	**0.01**

Note: Significant effects are shown in bold. Model: FiringRate~Area × day × interval + (1|Response) + (1|Neurons). Obs 3018772 Model R^2^ 0.15.

Next, we searched for neurons in which firing rates were affected by interval duration (Fig. 6*D*–*E*). Specifically, we used GLMMs to identify neurons with an effect of interval on firing rate (*P* < 0.05). Interval-modulated neurons were more common in the DMS than the MFC on Day 1 of two-interval performance (Fig. 6*E*; neurons with a main effect of interval on firing rate in one-way GLMMs; MFC 10 of 47 versus DMS: 28 of 58; X^2^ = 8.20; *P* = 0.004). Interestingly, ~ 50% of interval-modulated neurons also had ramping activity in MFC (5 of 10) and DMS (15 of 28). Of note, the number of interval-modulated neurons was not different between MFC and DMS on Days 2 and 3 (Fig. 6*E*). Consistent with these analyses, trial-by-trial GLMMs revealed a significant interaction between interval modulation, brain area, and Days 1–3 (three-way ANOVA of GLMMs of area, day, and interval on firing rate: *F*_(3018772)_ = 6.4, *P* = 0.01; Table 4).

Of note, we found in GLMMs that there was no main effect of response on firing rate (*F*_(3018772)_ = 2.5, *P* = 0.11), and no interaction with Days 1–3 (*F*_(3018772)_ = 0.4, *P* = 0.51). Taken together, these data indicate that while MFC ramping is sensitive to temporal context, DMS neurons increased temporal decoding for FI3 trials and had distinct interval-related modulations. These data provide insight into corticostriatal dynamics during temporal learning.

## Discussion

In this manuscript, we investigated whether MFC and DMS played similar or distinct roles in temporal learning. We found three novel distinctions between MFC and DMS during temporal learning. First, MFC but not DMS ramping decreased as animals that had been trained on a one-interval task learned to respond to a new temporal context that included a shorter second interval. Second, while MFC and DMS ramping was stable over 3 days of two-interval performance, DMS temporal decoding improved on the new FI3 interval. Finally, interval-modulated neurons were more common in the DMS early in two-interval performance. Our data suggest that MFC ensembles are sensitive to the context or “rules” of the task—that is, FI12 versus FI3/FI12, while the DMS optimizes behavior, particularly on FI3 trials. These data provide insight into the respective roles of prefrontal and striatal networks during temporal learning.

These results contradicted our hypothesis that MFC and DMS time-related ramping would be similarly affected by the introduction of a new temporal interval. Our hypothesis was based on 5 lines of evidence: 1) the existence of strong projections from the MFC to the DMS (Gabbott et al. 2005; Wall et al. 2013; Han et al. 2017), 2) clear roles for both structures in interval timing (Meck 2006; Coull et al. 2011; Emmons et al. 2017; De Corte et al. 2019), 3) similarities in time-related ramping in the MFC and DMS (Emmons et al. 2017), 4) the necessity of MFC activity for DMS ramping (Emmons et al. 2017, 2019), and 5) our recent demonstration that the stimulation of axons that project from the MFC to the DMS is sufficient to recover time-related ramping in the DMS (Emmons et al. 2019). Given these data, it is notable that the MFC and DMS play distinct roles during temporal learning, although this observation is concordant with the vastly different connectivity and synaptic organization of these two structures (Shepherd 2003). Decreases in MFC ramping after the introduction of a shorter interval suggest that MFC ramping is sensitive to the temporal context of the one-interval versus two-interval task, and they may reflect Bayesian priors of temporal probabilities (Jazayeri and Shadlen 2010; Shi et al. 2013). Our data are consistent with the theory that prefrontal ramping reflects learned “rules” (Wallis et al. 2001; Genovesio et al. 2005; Fuster 2008; Ruge et al. 2019), in this case, the temporal contexts of the one versus two-interval task. By contrast, striatal ensembles did not change on FI12 trials but improved temporal decoding on FI3 trials, suggesting a view that striatal ramping is related to optimizing performance (Pasupathy and Miller 2005).

Corticostriatal differences were anticipated based on a recent comparison of neuronal ensembles during a temporal categorization task that involved maze running (Kim et al. 2018). This study indicated that ramping was more prevalent in the MFC than the dorsal striatum. In this task, as the intervals became longer, temporal decoding by the MFC was less effective than that by the striatum. Although this task was more complex than ours and a number of others (Donnelly et al. 2015; Narayanan 2016; Bakhurin et al. 2017; Wang et al. 2018), the strong temporal encoding across corticostriatal ensembles is consistent with our findings here.

Our findings are in line with drift-diffusion models of two-interval tasks, as we find that time-related ramping scales with the interval duration (Simen et al. 2011; Xu et al. 2014). Here we find that MFC ramping is sensitive to temporal context whereas DMS ramping is not, and that nonramping interval-related modulations and temporal predictions in the DMS change with two-interval performance. These results suggest that time-related ramping reflects distinct processes in MFC and DMS. Given that MFC activity influences ramping in DMS (Emmons et al. 2017, 2019), DMS ramping activity might integrate aspects of MFC ramping as well as nonramping activity.

Because time-related ramping activity in MFC and DMS ensembles did not change during two-interval performance, ramping activity may be remarkably stable in both brain regions when the temporal context does not change. It is unclear how ramping might change with extended periods of behavior over several days or weeks (Barnes et al. 2005; Yin et al. 2005; Graybiel 2008). However, we did find that on FI3 trials, temporal decoding in the DMS improved even though DMS ramping was stable. In the DMS, patterns beyond ramping activity might change during two-interval performance and contribute to improved temporal decoding (Paton and Buonomano 2018). The improvement in temporal prediction despite unchanged ramping activity supports improved “population clock”-based temporal predictions during FI3 trials (Karmarkar and Buonomano 2007; Laje and Buonomano 2013). Finally, <15% of corticostriatal neurons can be modulated by responses, and this modulation can interact with ramping activity (Emmons et al. 2017). However, in the present dataset, we found that response-related activity did not change over subsequent days of two-interval performance, and that there were main effects of context even when responses were matched between Day 0 and Days 1–3. These data imply that the shifts in MFC ramping and DMS temporal decoding could not be fully accounted for by changes in responding.

We found that the DMS contained more neurons in which there was a main effect of interval compared to the MFC on the early days of two-interval performance. Notably, half of interval-modulated neurons were not ramping. These data suggest that patterns beyond time-related ramping encode information about temporal intervals. On progressive days of two-interval performance, interval-related activity between the MFC and DMS equalized. Because our task design involved a second cue for FI3 intervals, we cannot distinguish whether this activity was related to working memory for temporal intervals, cue-related processing, or other aspects of interval timing. Future work using more advanced learning paradigms may clarify these patterns of activity.

To our knowledge, our study is one of the first to record from corticostriatal ensembles during temporal learning and to suggest that components of corticostriatal ensembles play distinct roles in temporal learning. The striatum has a well-established role in learning other contexts including habit formation, reversal learning, and instrumental learning (Yin and Knowlton 2006; Kimchi and Laubach 2009; Graybiel and Grafton 2015). Striatal neuronal ensembles robustly encode time, and this temporal encoding rapidly rescales as animals learn novel intervals (Mello et al. 2015). While prefrontal ensembles are also involved in learning (Baeg et al. 2007), direct comparisons with striatal ensembles during learning are rare in rodents. One exception is a recent study of prenatal alcohol exposure, which showed that the orbitofrontal cortex disengages and the dorsal striatum updates reward contingencies (Marquardt et al. 2020). These findings parallel the changes in the MFC and DMS that we report here. The observation that prenatal exposure to alcohol leads to changes in cortical activity underscores the clinical significance of this brain circuit.

During associative learning in primates, corticostriatal ensembles are highly sensitive to learning, with striatal neurons rapidly encoding new associations and the prefrontal cortex learning more slowly (Pasupathy and Miller 2005). Primate striatal neurons rapidly encoded stimulus-response associations, whereas primate prefrontal neurons encoded category abstraction (Antzoulatos and Miller 2011). In line with these results, we found that time-related ramping in prefrontal regions decreased in two-interval versus one-interval sessions, suggesting that these ensembles may be sensitive to temporal categories or context. It is important to note that these primate studies recorded from lateral prefrontal areas, which lack a clear rodent analogue (Laubach et al. 2018), and that they employed vastly different task conditions. Furthermore, more recent work has shown that prefrontal ensembles can rapidly change during learning (Schuck et al. 2015; Siniscalchi et al. 2016), and therefore, the relative changes in corticostriatal ensembles may depend on the details of each task. Prior work has consistently established that corticostriatal neuronal ensembles are modulated during interval-timing tasks (Matell et al. 2003; Bakhurin et al. 2017; Emmons et al. 2017) and shown that single neurons in the prefrontal cortex (Xu et al. 2014) or striatum (Portugal et al. 2011; Mello et al. 2015) can flexibly adapt to new temporal contingencies. Our data are congruent with this work and directly compare MFC and DMS, suggesting that MFC ramping is sensitive to temporal context while DMS ensembles increase temporal decoding on trials with a novel, shorter interval. Our work provides insight into the dynamics of rodent corticostriatal ensembles during an elementary temporal learning paradigm.

Our study has several limitations. First, we used fixed-interval timing; peak-interval timing tasks might enable more precise dissection of start and stop times (Buhusi and Meck 2005). Indeed, the effects reported here may extend beyond timing processes and could also be a result of temporal discrimination of FI3 trials, learning a new contingency on FI3 trials, or disrupting an existing contingency on FI12 trials. In addition, we did not counterbalance between cues for FI3 versus FI12 trials, which would not affect comparisons of FI12 or FI3 trials over days (Figs 3–5), but might affect comparisons between activity on FI12 and FI3 trials. However, animals’ response times on both FI12 and FI3 trials were responsive to both temporal contexts over subsequent days of two-interval performance. Despite stimuli and training differences between FI3 and FI12 trials, both responses and corticostriatal neuronal activity were affected by adding the FI3 interval. Our neural findings strongly suggest dynamic roles for corticostriatal ensembles during interval timing. Second, we did not track individual neurons over days. We describe MFC changes to temporal context and DMS changes over two-interval performance at group-level analyses (i.e., PC1 and decoding) as well as by trial-by-trial analyses of firing rate (Tables 2 and 4)—thus, our data provide insight into flexibility among corticostriatal ensembles rather than at the single-neuron level. Third, our techniques cannot identify the genetic or molecular identity of recorded neurons. This detail would be of particular interest in the case of the DMS, which contains D1 and D2 MSNs, which play complementary roles in movement (Kreitzer 2009). Fourth, we are unsure if the MFC and DMS neurons that we captured were synaptically connected, because of the sparsity of cortical projections and constraints of our recording techniques (Wall et al. 2013). This limitation might be overcome in future work by exploiting optogenetic tagging and retrograde viral tracing to isolate corticostriatal projections (Otis et al. 2017). Studying how MFC-DMS connectivity changes with learning might provide further insight into corticostriatal circuits. Fifth, we were unable to identify clear behavioral correlates of temporal learning during the first two-interval session on Day 1. Behavioral transitions can be faster adjusting to shorter intervals and slower adjusting to longer intervals (Higa 1996, 1997; Higa and Tillou 2001). Furthermore, adapting to new intervals may require multiple adjustments (Meck et al. 1984). In addition, FI3 trials involve a higher reward rate as more rewards are acquired on average per second. Our data cannot address these issues and are instead focused on a single transition—from sessions with FI12 to sessions with FI12 and FI3 trials—but future work will be required to explore how corticostriatal ramping adapts to longer intervals and to distinct reward rates. Note that two of our main points—that MFC ramping decreases on FI12 trials, and that DMS temporal decoding increases on FI3 trials—were derived from comparisons of similar trial types, and were not affected by differences in training history, reward rate, and other features that differ between FI12 and FI3 trials. Corticostriatal ensembles may rapidly learn the new FI3 interval. The striatum is essential for rapid adaptations (MacDonald et al. 2012), but capturing changes in MSN firing might require a different task design to capture trial-by-trial neuronal dynamics during rapid learning (Kimchi and Laubach 2009).

In summary, we investigated whether the MFC and DMS played similar or distinct roles in temporal learning. We recorded from corticostriatal ensembles in rodents that had been trained to perform a single fixed-interval timing task while they learned to incorporate a new interval. We discovered that for FI12 trials, time-related ramping activity in the MFC decreased following introduction of the shorter interval, whereas ramping activity in the DMS was unchanged. For FI3 trials, we found that corticostriatal ramping activity did not change, yet DMS temporal decoding improved. Finally, more DMS neurons fired differentially on each interval compared to the MFC early in two-interval performance. Taken together, our data provide novel evidence that the MFC and DMS play distinct roles in temporal learning.

## Notes

We thank Morgan Kennedy and Tomas Lence for technical help. E.E., Y.K., and N.N. designed experiments; E.E. and G.T.-C. collected data; E.E., Y.K., J.C., and N.N. analyzed data; A.B. independently checked the code and data; and E.E., G.T.C., M.W., A.B., Y.K., and N.N. wrote the manuscript. Data and code available at narayanan.lab.uiowa.edu. *Conflict of Interest:* None declared.

## Funding

National Institutes of Mental Health R01 fund to N.N. and Titan Neurological Fund to N.N., National Institutes of Mental Health F31 fund to E.E., Coordination for the Improvement of Higher Education Personnel fund to G.T.-C.
